# An Unusual Presentation of Nodular Hidradenoma

**DOI:** 10.7759/cureus.44897

**Published:** 2023-09-08

**Authors:** Sandhya R Palit, Vidhya Sree S, Nikhithaa P, Krithi Sree S, Viswanathan M S

**Affiliations:** 1 General and Colorectal Surgery, Employees' State Insurance Corporation Medical College (ESICMC) and Postgraduate Institute of Medical Sciences and Research (PGIMSR), Chennai, IND; 2 General Surgery, Employees' State Insurance Corporation Medical College (ESICMC) and Postgraduate Institute of Medical Sciences and Research (PGIMSR), Chennai, IND; 3 General and Plastic Surgery, Employees' State Insurance Corporation Medical College (ESICMC) and Postgraduate Institute of Medical Sciences and Research (PGIMSR), Chennai, IND

**Keywords:** apocrine and eccrine, apocrine differentiation, dermal tumours, benign tumors, clear cell hidradenoma, nodular hidradenoma

## Abstract

Benign adnexal neoplasms are quite a common occurrence in adults, especially in the head and neck region. They raise suspicion for malignancy if there are red flag signs like rapid increase in size, pain, ulceration or recurrence. We hereby report a case of a middle-aged gentleman who consulted our surgical OPD with right-sided neck swelling, which was initially thought to be a dermoid cyst; on further evaluation, found to be a dermal sweat gland tumour with features of nodular hidradenoma. The point that is of interest but coincidence to note is that this swelling was preceded by a minor trauma.

Characterising these swellings using simple imaging and pathological investigation modalities is important to study their behavioural pattern and add the same to our existing database. This will also help the treating surgeons to keep in mind the possibility of occurrence of such histologies in soft tissue swellings when they present with uncommon clinical features, instead of brushing them aside as the common epidermal or dermoid cysts. Incidence of malignancy is almost nil in nodular hidradenoma, which when found, is attributed to poor surgical clearance; hence the prudence to operate with adequate clearance is extremely significant in preventing the transformation of a mole into a mountain.

## Introduction

Nodular hidradenomas or clear cell hidradenomas are asymptomatic, benign, nodular intradermal tumours that make up 95% of all hidradenomas [1]. Although they were previously thought to exhibit eccrine differentiation, it is now found that these tumours can exhibit either eccrine or apocrine differentiation [2,3]. Hidradenomas are mostly found in adults, with an increased female preponderance. Incidence in children is very rare [4].

They usually present as well-defined and multilobulated tumours seen only in the dermis or in both the dermis and the subcutaneous tissue. The primary pattern is mostly solid, the other is both solid and cystic, and occasionally tubulocystic structures are observed. These tumours are slow-growing and are usually present for many years before diagnosis [5].

In rare cases, malignant transformation of clear cell hidradenoma with metastatic spread has been reported [6].

The point of interest but coincidence in our case is that the swelling was preceded by a minor trauma. Owing to the patient's clinical signs and symptoms and history, a preliminary diagnosis of implantation dermoid cyst was made. But with further investigation, it was found to be nodular hidradenoma.

## Case presentation

**Figure 1:**
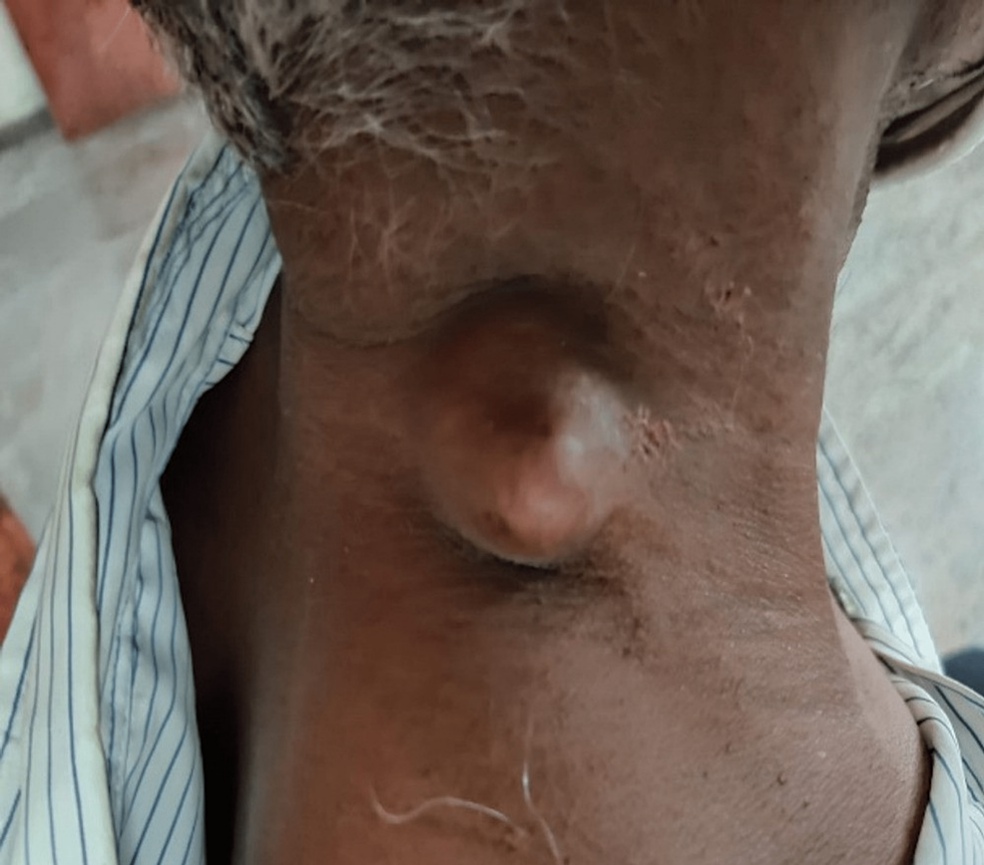
The patient at the time of presentation with a swelling over the right posterior triangle of the neck

**Figure 2:**
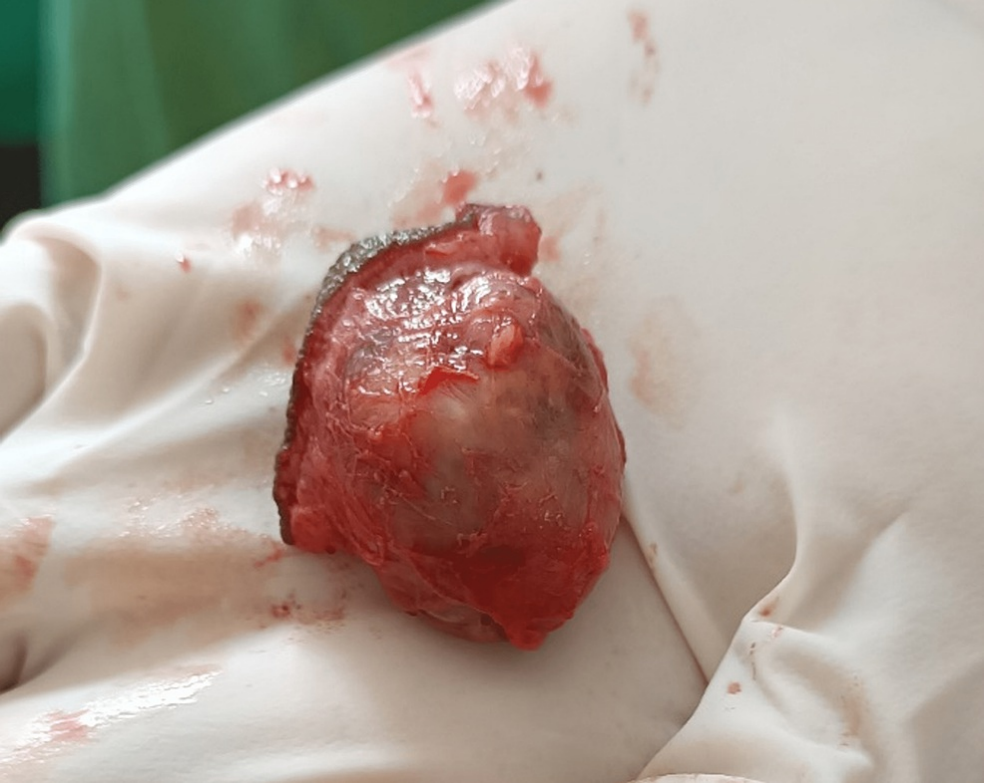
Swelling excised in toto

**Figure 3:**
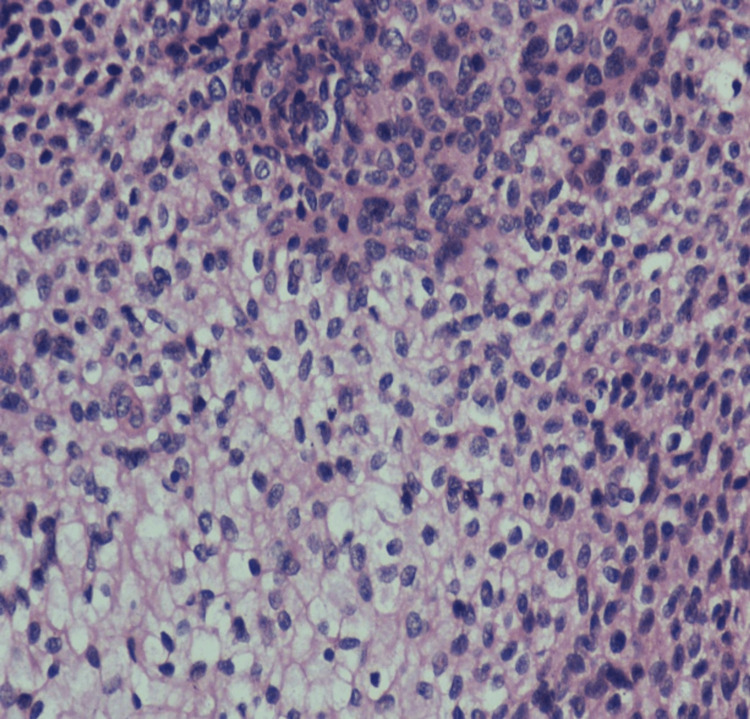
H&E staining showing round to oval cells with round nucleus and clear cytoplasm

A 48-year-old man presented with a swelling on the right side of his neck that had been present for the past two months. The patient reported that the swelling developed after a pin prick injury. Upon examination, a firm, smooth, and freely mobile swelling measuring 3x3 cm was observed on the right posterior triangle of the neck. The swelling was firm, non-tender with a smooth surface; had a fluctuant summit; freely mobile and the skin overlying the swelling was pinch-able except over the summit (Figure 1). Based on the clinical findings, a preliminary diagnosis of implantation dermoid cyst was made, which was later confirmed by imaging. Ultrasound showed a 4x3 cm ovoid heteroechoic lesion, with mixed solid and cystic areas, without any increased vascularity. To further characterize the lesion, a fine needle aspiration cytology (FNAC) was performed, which revealed an adnexal lesion, arising from the appendages of the dermis. The patient was taken up for excision under local anesthesia after obtaining informed consent. An elliptical incision over the summit was made, deepened, meticulously dissected and the swelling excised in toto (Figure 2). A wide local excision was done with a 0.5 cm gross margin under local anesthesia with 2 percent lignocaine (dosage calculated according to the patient's weight) and was infiltrated around the swelling. The excised swelling was examined and found to be a well-defined, lesion located in the dermis. On histopathological examination, it consisted of both solid and cystic areas. The cystic areas contained tubular lumina lined by a single layer of cuboidal epithelium, while the solid areas showed sheets of round to oval cells with round nuclei and clear cytoplasm (Figure 3). These features indicated a nodular hidradenoma. The patient was discharged and followed up on OPD basis. Sutures were removed on the 7th postoperative day. Then he was followed up for three months till the end of the first year, which was uneventful without any recurrence. The patient was then advised to review after three months and continue to do so till the end of the second year.

## Discussion

Hidradenomas are benign skin adnexal neoplasms that are rare and encompass a wide range of differentiation and most commonly occur in adults. The exact incidence rates of nodular hidradenomas have not been characterized; malignancy is reported to be less than 0.001% in various studies. They present as solitary tumours that are generally firm but freely mobile [7]. They are also progressively enlarging tumours which may have a pedunculated or cystic appearance. While most exhibit eccrine differentiation, apocrine differentiation is not completely absent either [8]. The average size is reported to be 0.5 to 2 cm. There have been cases with nodules as large as 15 x 7.8 x 5 cm, according to a report published in Pathology International in 2008 [9]. It is mostly seen in patients who are aged beyond 6th decade and in rare instances, seen in children as well [10]. Usually, the nodule appears in the head and neck region, and in the extremities [11]. The skin overlying the nodule is generally intact, but sometimes serous discharge and ulceration may be seen [12].

The histology of a nodular hidradenoma can be described as a lobulated mass that is well circumscribed which mainly extends to the dermis and sometimes, can spread subcutaneously [13]. Two cell types can be seen, one type which is small with clear cytoplasm with eccentrically placed nuclei, or normal-sized round or polygonal cells with eosinophilic cytoplasm with oval vesicular nuclei [14]. The tumour often has the presence of transitional cells. Further, the presence of tubular or ductal structures is noted that are of wide range in size and in number [8]. The presence of decapitation secretion may be a sign of apocrine nature of the tumour cells. In most cases this feature is generally absent or focal [15]. Both squamous and sebaceous differentiation are common, with squamous being more frequently seen with infundibular type of keratinization. Both hyaline and myxoid variants of stroma are conventionally seen [8].

The tumour cells generally express AE1/AE3, EMA, and CEA [11]. A translocation involving MAML2 gene can also be evidenced by fluorescence in situ hybridization (FISH) analysis [12]. But immunohistochemical analysis is not routinely done, and eccrine tumours are diagnosed with hematoxylin-and-eosin-stained sections alone [7]. In our case, a well-circumscribed benign lesion in the dermis was composed of solid and cystic areas; the cystic areas contained tubular lumina lined by a single layer of cuboidal epithelium, and the solid areas showed sheets of round to oval cells with round nucleus and clear cytoplasm was seen. Epidermal attachment is usually uncommon, but sometimes between the tumour and the epidermis, a Grenz zone may be seen [16]. This was not seen in our case. The clear cells seen in histopathology, as in our case, can be due to glycogen accumulation in large amounts, but not lipids, which is Periodic acid-Schiff (PAS)-positive [8].

It is generally a slow-growing tumour. On the other hand, if it progresses quickly, it may be a sign of malignancy [17]. Since, in our case, the tumour was very slow growing, the suspicion of malignancy was quite low. Malignant hidradenoma exhibits histological features that closely resemble its benign counterpart. However, there are distinctive criteria that indicate malignancy, such as a lack of well-defined borders, the presence of abnormal nuclear characteristics and increased cellular division, predominantly solid cell clusters, an infiltrative growth pattern, regions of tissue death, and infiltration into the blood and lymphatic vessels [18].

The precise incidence rate of transformation to malignant hidradenoma has yet to be definitively established. However, it remains an elusive figure within the current body of knowledge. Notably, malignant hidradenomas exhibit an elevated expression of PHH3 greater than 0.7% and/or Ki-67 exceeding 11%. These markers serve as indicators of increased cellular proliferation and activity, further underscoring the complex nature of malignant transformation in hidradenoma [7].

Emphasising the significance of comprehensive surgical intervention with adequate margins is paramount in mitigating the risk of local recurrence. The optimum margin is when the margins are clear of the tumour both macroscopically and microscopically. Wide surgical excision, encompassing an appropriate margin of healthy tissue surrounding the lesion, is crucial in ensuring complete removal of nodular hidradenoma and reducing the likelihood of its resurgence in the local area [7]. By employing this meticulous approach, surgeons aim to minimize the possibility of residual tumour cells remaining after excision, thereby optimizing the long-term outcomes and reducing the potential for local recurrence.

Nodular hidradenoma recurrence is common, occurring in up to 10% of cases. This high recurrence rate is largely attributed to inadequate excision of the tumour during the initial surgical intervention [19]. Close monitoring and frequent clinical assessments are crucial in order to detect any potential recurrence or malignant changes at the earliest stages [9]. This enables timely intervention and the implementation of appropriate management strategies, ultimately improving the overall prognosis and ensuring optimal patient outcomes.

## Conclusions

Nodular hidradenomas, also known as clear cell hidradenomas, are predominantly asymptomatic and benign nodular tumours located within the dermis. They have a slow growth rate and can go undiagnosed for many years. These tumours often remain asymptomatic and may not be noticed until they reach a significant size or cause noticeable changes. Achieving meticulous and comprehensive excision of nodular hidradenoma is of utmost importance in order to minimize the risk of recurrence and optimize long-term patient outcomes.
